# Discussion of Present Status of the American Institute

**Published:** 1885-02

**Authors:** P. P. Wells

**Affiliations:** Brooklyn


### HOMŒOPATHIC PHYSICIAN.

A MONTHLY JOURNAL OF MEDICAL SCIENCE.

"If our school ever gives up the strict inductive method of Hahnemann, we are lost, and deserve only to be mentioned as a caricature in the history of medicine."—CONSTANTINE HERING.

Vol. V. FEBRUARY, 1885. No. 2.

### DISCUSSION OF PRESENT STATUS OF THE AMERICAN INSTITUTE.

DEAR HOMŒOPATHIC PHYSICIAN:—The following is *part* of a correspondence of a recent date between a leading member of the American Institute and myself. I regret that my correspondent makes giving it entire impossible, by refusing his consent to publishing his letter of December 1st, in connection with my reply to it. I can only give brief extracts from it, which have served as basis for my reply, and may be useful as stepping-stones to a standpoint from which the views of the writer, and presumably those of the American Institute, may be clearly seen. Thus arranged, the correspondence will explain itself.

BROOKLYN, December 2d, 1884.

DEAR DOCTOR:—I have yours of the 1st inst. In some of the matters it treats we differ in knowledge or judgment, or perhaps both. If, as you say, you have read my articles in THE HOMŒOPATHIC PHYSICIAN, you may remember that some portions of your letter are in these answered before its reception. If you will turn to the number for December inst., you will find in it as plain a statement as I can make of my views of the present character of the American Institute of Homœopathy, and also of the character of its work, and you may there find a small part of the evidence it has given to the world that it has ceased to be an institute of *Homœopathy* at all. You say of me that I "have abandoned the American Institute of Homœ-

opathy." This is a mistake. It is the other way, as you may learn from the paper referred to: It is the Institute which has abandoned Homœopathy. Beyond retaining its name, I fail to find aught in its doings or opinions expressed the last few years belonging to Homœopathy at all. And this is all the difficulty I have with it. I have elsewhere spoken plainly on this subject. There can be no need of repeating this to you. You have read it. If there be any answer to this, disproving what I have said, or any showing that I am mistaken, it will be a pleasure to me to see it. If I have spoken the truth, let the Institute mend its manners and its ways; let it return to labors in the interest of true Homœopathy, and I am content; let it cease its efforts to destroy, and return to such as will build this up, and it shall have from me only my best wishes.

"The I. H. A. is doing no good, nor will it do any, so long as irrational and unscientific posology is encouraged.

In regard to the I. H. A. and its usefulness, I may say I never favored its organization, never entertained any very exalted expectations of its usefulness. But I cannot shut my eyes to the fact that its existence is a protest against the abandonment of homœopathic law and philosophy by the American Institute of no small significance. Its existence is a rebuke of this abandonment of no mean power. The I. H. A. had its origin in a deep, indignant sense of this abandonment. It was from this, and not from any peculiar views of posology, that the I. H. A. had its origin.

"Fluxion potencies are a laughing stock to all right-minded men."

You are mistaken. There are not a few men who seem right-minded, who, after practical experience of these potencies, hold them in high esteem, and this because of witnessing their successful use in clinical cases after the failure of other preparations to relieve in the same cases. There are many such men, who have come to their general use because they have found them more potent curatives than other preparations. What other possible motive could they have for adopting them into their practice? And are not "right-minded men" called on to accept their testimony, that of these men who have tried them and know, rather than to heed the silly laughter of the American Institute of Homœopathy, the laughter of those who have *not* tried and do *not* know? Doctor, I have no doubt I *know* more of these potencies than you do. If you had *known* them as I *know* them, you could never have spoken of them as you have, if you are but half as honest minded as you ought to be, and as

I have given you credit for being. It is only because you don't know that you call them "irrational," and because the silly Institute doesn't know that it laughs. Fluxion potencies are not going to "ruin our school"—be at perfect ease on this head. I wish I could say as much as to abandonment of homœopathic law and philosophy. If our school is to be ruined, you and I, if we live to see it and see clearly the cause of the "ruin," will surely find this abandonment the most efficient cause in bringing about this result, and the American Institute its most active agent.

"It is not successful [practice with fluxion potencies] as compared with sound and moderate posology," etc.

What is "sound" and what is "moderate" in posology? Define these words, and then we may be able to see how far the statement is correct. That is most "sound" in posology, we take it, which cures sickness in greatest proportion, in shortest time, with greatest certainty and safety. And it is affirmed of these potencies by those *who know* that, as compared with other preparations, this is just what they do. If their testimony be true, it is not easy to see just how they are to bring about the foreboded "ruin." It is not difficult to see how abandonment of principles may do this. Indeed, there are those who think they see just how this is *now* doing it.

"Pure Homœopathy is bound to survive, and that on a sound and scientific basis."

It is to be hoped that it will, but here again we need definitions. "Pure," "sound," "scientific." If by "pure" be meant law without mixture of men's fancies, good. "Sound"—well, we don't quite so easily come at that. But if it means that which an enlightened experience has established as best, good again. "Scientific." This is easy. It is predicated of *Homœopathy*. This, as we know, is the *science* of therapeutics, and there is no other. This, then, can only mean a practical therapeutics founded on law. So, and so only, can its foundation be "scientific." A therapeutics founded on something else, in whole or in part, can never be a "scientific" therapeutics. So founded, and a practice based on it is ever and only a practice in the dark.

If this be so, dear Doctor, then does it throw no light on the duty of those who have accepted the responsibility of teaching a therapeutics based on God's law? And does it not present in dark shadow the just judgment those incur who neglect so to teach?

4

"It has been laughed out of the American Institute," etc.

That is, cures by fluxion potencies have been so treated. And this reminds me that there is a man somewhere out West, known as "Bob Ingersoll," who has been trying these many years to laugh the God of Heaven from His throne. I do not think he has had much success in his folly, though perhaps he is satisfied with his endeavor. Suppose he had succeeded? It would only have been the worse for poor Bob as well as the rest of mankind. Just so with this truth the Institute is said to have laughed out of their sight. It does not hurt the truth at all, and I cannot help thinking it would have been better for them if they had permitted themselves to be instructed by this truth instead of indulging in untimely laughter. Truth is truth, after all their laughter, the same as before, great as this may have been. Truth, dear Doctor, is stronger than laughter, and don't forget it.

P. P. WELLS.

### No. II.

BROOKLYN, December 6th, 1884.

DEAR DOCTOR:—I have yours of the present date, withholding your assent to the publication of yours of the 1st inst. with my reply to it. I regret this a little, as it expresses so much of what I regard as characteristic of the present status of the American Institute, and coming from a representative member of the Institute, it seemed to me more of a declaration of that status than a mere private communication from one individual to another. Its chief interest was rather of a public than of a private nature, and as my reply was as clear a statement as I could make of my views of this matter of public interest, good might result from their going together before the public, who have the chief concern in the matter. I have read both to a few judicious friends, who have expressed a decided opinion that I have a perfect right to publish both without your consent, but I should greatly have preferred to do it with. If I conclude to print, I will withhold your name, so you shall not be held before the public as in any way connected with, or responsible for, the writing or printing of either. It seems to me well that the two sides should go together to the public, and that now is as good a time as any for these utterances.

Your letter of this morning reads like a plea of guilty on behalf of the American Institute to my indictment of that body for having abandoned Homœopathy. You say:

"I think I know the temper of a large majority of the Institute, and it is materialistic in medicine."

Just so. And this is my difficulty with the Institute. Here is just where the *abandonment* is found. The Institute is materialistic. This is just what Homœopathy is not. Homœopathy is dynamic in its nature, in its philosophy of its objectives—the cures of sicknesses—and of the means it employs for these cures. It is dynamic in whole and in part. And hence the folly of those who would pursue investigations in this field by methods and means employed in experiments and investigations in physics. They are looking for fruits in one field which only grow in another, and it may be in one far away. The materialist, dear Doctor, has nothing to do with God's Homœopathy, nor can he have, except—and sad it is to say it—he sometimes most unjustifiably usurps its honorable name.

To attempt investigations of homœopathic philosophy and practice by the means and methods employed in researches in physical science is as absurd as to attempt research in astronomy by the means and processes of the chemical laboratory, or researches in mental philosophy by the same means. The science of therapeutics (Homœopathy) is a science *sui generis*, and not dependent on other or physical sciences for its existence or for means necessary to its right understanding or investigation. It can only be investigated by means adapted to its own nature—*i. e.*, to a science of dynamics. To attempt this by the microscope, crucible, or test-tube is only a braying of the animal who attempts to act the part of the lion, having only his skin.

"Homœopathy is a reform, * * * * and must establish and fortify its position."

Homœopathy, dear Doctor, is not on trial before the American Institute or before any man. It had its trial before you were born. The judicature was sick humanity, and the verdict was in its favor. There is no call, that I know of, for a new trial, either on the part of Homœopathy or sick mankind, and these are the parties chiefly interested. The call for the new trial is altogether from those who have abandoned God's science of therapeutics, and therefore have no interest in the matter which can give them a standing in court. They are only interested and busy in endeavors to set aside this honorable world's verdict, and in efforts to replace in the professional mind the dynamic nature of homœopathic philosophy and means with the musty, foggy materialism of old-school physic. Not a very honorable employment, and not likely to succeed in the end. There will be no new trial, for none is needed. There may be a trial of these abandoners, and then a better state of mind and knowledge may possibly be attained by them.

"* * * * I am sure Homœopathy never needed defense more than now."

Homœopathy is suffering chiefly from the misdoings and utterances of the American Institute. It is to be hoped it will survive these by reason of the truth which is in it. It only needs "*defense*" from attacks of those who bear its name and pretend to be its friends. Is this "*defense*" too much to ask and expect from yourself and other "right-minded men" who are really desirous of the best interests of Homœopathy?

"While the strong men of our school adhered to the *Organon* and used and reported cures with Hahnemann's potencies, the school advanced; but it seems to me that the change has come about with the introduction of Fincke's fluxions and their adoption by Hering, Lippe, Guernsey, etc. Dunham was too wise to indorse them."

Dunham was wise enough to purchase and use them. It is altogether too common just now, here and elsewhere, to call the name of this honored and lamented colleague to the support of all kinds of error or opinions to which, not unfrequently, he had never given his sanction. This is a safe dodge for the advocate of whatever is untenable in opinion or an error in practice, as Dunham can no longer speak for himself. But who were "the *strong* men of our school" if we are to class Hering with the *weak?* Surely this is putting Fincke's fluxions into good company; and we may be permitted to remark right here, that it might have been better if those of our school not so "strong" as Hering had permitted themselves to be instructed by the example of this "noblest Roman of them all," instead of posing as sneerers at an experience they seem so incapable of comprehending or imitating. Doctor, allow me to speak plainly to you my judgment as to that which to you seems to need defense.

It is not, I think, Homœopathy. That needs none. It is only the feebleness, ignorance, and short-comings of men who claim to be of our school, and honestly think they are, while there is little of it in or of them except its honored and abused name. How comes it that these are now so numerous that you judge them to constitute a majority of the membership of the American Institute? The cause, I think, is not far to seek. If you will turn to a recent number of THE HOMŒOPATHIC PHYSICIAN (August, Vol. IV), you will find a paper on "Medical Education, and Our Colleges as Contributors Thereto," in which these colleges are held responsible for the materialism of the younger members of our school. These have come out of the schools, not having been taught dynamism, but with so full a charge of materialism as to seem to have no consciousness that there is, or can be, anything besides matter with which they, as professed practitioners of *specific* medicine, have, or are to have, anything to do. Where have these young members learned this materialism, which is so far away from all of Homœopathy, except in these colleges? And are we wrong if we charge on these the origin, through these juniors, of all that which you say needs "defense," and of all that part of the history of the American Institute which has so greatly disgraced itself and the school it is claimed to represent? The "change" you deprecate has "come about"—not from "*fluxion potencies*," but from *these colleges* and the wrongly educated men whom they have sent out to practice a system of specific medicine, the philosophy of which they have never heard, and of which most of them are so profoundly ignorant.

"What earnest seeker after truth, with a desire to convince the skeptical, would undertake to use such methods?" * * * [*i. e.*, the methods of preparing and using fluxion potencies.] "They are so improbable to a student of physics, and so infinitely transcendental, that to be adopted they should support their claims by overpowering and unmistakable results."

This may be so to a "student of physics," but how to a student of dynamics, with which alone these potencies have to do? The "student of physics" has no concern with them. They are not of his domain, and are not amenable to his methods and processes—not even to the microscope, crucible, or test-tube.

"They should support this claim (that for these potencies of curing power) by overpowering and unmistakable results. These have not been forthcoming."

That these potencies cure is proved by the same evidence in kind and amount as proves the same fact of the third or the thirtieth or any other number of a potentized drug, or of any amount of any crude drug. They are given in a case of sickness, with a given group of phenomena, accompanied by certain sufferings, and these phenomena and these sufferings cease. This is all the proof of curing power these or any other form of medicine *can* give. And, we submit, no other is or can be needed. He who demands more, or more for these potencies because they are not of the domain of physics, has himself gone beyond the domain of reason. These potencies have been given to the sick, and the sick have been cured by them, with a uniformity at least equal to that which follows the use of any other form of drug administration, for *more than twenty years*. They have been given to innumerable cases, many of which have been reported and published, and as fairly and plainly as have cures by any form of drug agency. This has been done just as other reports of cures have been given from other drug agencies which have been attested as evidence of curing power in these agencies. Then, why not accept the same kind of evidence of curing power in these fluxion potencies? Is not the "overpowering" and "unmistakable" found in these experiences? If not, why not? Is the claimant for the "overpowering" demanding some kind of an argumentative earthquake power? Well, we admit fluxion potencies do not deal in this.

And now, Doctor, one word as to skeptics and skepticism. There are two kinds of this in the world, one of the reason and one of the will. Defect of knowledge may be cured by bringing before reason evidence of facts logically related to the matter in hand. By acceptance of this evidence reasonable skepticism is cured, and there is the end of it. Not so the skepticism which is of the will. Neither evidence, logic, nor truth, of any amount or of any character, have power to overcome this. These are rejected by a perverse will, which persists in unbelief in spite of these. Nothing short of the power which created will can carry light and truth into a human mind so guarded against them. There is much of this kind of skepticism in the world, more than of that which comes from want of evidence of truth. It would seem that skepticism as to the curing power of fluxion potencies after these twenty years of accumulated evidence of the fact, which is now daily added to, must be of this incurable kind—that of the *will*, and not of the *intellect*. There is only *one* "crucial test" of this claimed fact, and that is in the affirmative or negative of the question—*Do they cure?* We have no hesitation in saying, because we have *seen* it, they both cure and *make sick*.

P. P. WELLS.

---

THERAPEUTIC HINTS.—LOBELIA—Extreme *tenderness* over the sacrum. She cannot bear even the pressure of a soft pillow. She cries out if any attempt is made to touch the part. She sits up in bed, leaning forward to avoid contact with bed.

LOBELIA.—After each vomiting spell, she breaks out all over with *sweat*, followed by a sensation as if *thousands of needles* were piercing her skin from within outward.

CHELIDONIUM.—Dry cough through the day, with pain and stitches in *right* side, with severe *hoarseness* each evening at five o'clock, so that her voice could scarcely be heard.—C. CARLETON SMITH, M. D.

